# Microglial p38α MAPK is critical for LPS-induced neuron degeneration, through a mechanism involving TNFα

**DOI:** 10.1186/1750-1326-6-84

**Published:** 2011-12-20

**Authors:** Bin Xing, Adam D Bachstetter, Linda J Van Eldik

**Affiliations:** 1 Sanders-Brown Center on Aging, University of Kentucky, Lexington, KY 40536 USA; 2 Department of Anatomy and Neurobiology, University of Kentucky, Lexington, KY 40536 USA

**Keywords:** microglia, cytokines, knockout mice, p38alpha mitogen-activated protein kinase, neuron, tumor necrosis factor alpha

## Abstract

**Background:**

The p38α MAPK isoform is a well-established therapeutic target in peripheral inflammatory diseases, but the importance of this kinase in pathological microglial activation and detrimental inflammation in CNS disorders is less well understood. To test the role of the p38α MAPK isoform in microglia-dependent neuron damage, we used primary microglia from wild-type (WT) or p38α MAPK conditional knockout (KO) mice in co-culture with WT cortical neurons, and measured neuron damage after LPS insult.

**Results:**

We found that neurons in co-culture with p38α-deficient microglia were protected against LPS-induced synaptic loss, neurite degeneration, and neuronal death. The involvement of the proinflammatory cytokine TNFα was demonstrated by the findings that p38α KO microglia produced much less TNFα in response to LPS compared to WT microglia, that adding back TNFα to KO microglia/neuron co-cultures increased the LPS-induced neuron damage, and that neutralization of TNFα in WT microglia/neuron co-cultures prevented the neuron damage. These results using cell-selective, isoform-specific KO mice demonstrate that the p38α MAPK isoform in microglia is a key mediator of LPS-induced neuronal and synaptic dysfunction. The findings also provide evidence that a major mechanism by which LPS activation of microglia p38α MAPK signaling leads to neuron damage is through up-regulation of the proinflammatory cytokine TNFα.

**Conclusions:**

The data suggest that selective targeting of p38α MAPK signaling should be explored as a potential therapeutic strategy for CNS disorders where overproduction of proinflammatory cytokines is implicated in disease progression.

## Background

Extensive evidence, both clinical and preclinical, implicates neuroinflammation and overproduction of proinflammatory cytokines as a contributor to pathophysiology of chronic neurodegenerative disorders such as Alzheimer's disease (AD), Parkinson's disease, and multiple sclerosis [for review, see: [1]]. Proinflammatory cytokine overproduction has also been documented as detrimental to recovery in acute brain injuries such as trauma or stroke [2-5]. In the brain, activated microglia are a major mediator of neuroinflammation and can release a number of potentially neurotoxic substances, such as reactive oxygen species, nitric oxide, and various proinflammatory cytokines, of which two main proinflammatory cytokines TNFα and IL-1β are generally considered primary mediators leading to neurotoxicity [for detailed reviews on microglia, see: [6,7]].

There are many critical roles for innate immunity, and thereby the primary effector cells, microglia, in the classically immune privileged CNS. For example, microglia are rapid responders to local tissue stressors [8,9], can efficiently clear apoptotic cells during neurodevelopment [10], and can promote neuro-repair through the production of growth factors [7]. The spectrum of activated microglia phenotypes is diverse and generally beneficial. It is only when the activation becomes exaggerated or dysregulated does the response become neurotoxic. Therefore, it is of critical importance to elucidate the mechanisms that are specifically involved in the dysregulated response of microglia which contribute to neuronal damage.

Intracellular signal transduction cascades regulate the production of proinflammatory cytokines. By targeting a specific signal transduction pathway it is possible to determine if a pathway is involved in the dysregulated response that is neurotoxic and if the dysregulated response is amenable to intervention. One of the most well established signal transduction cascades that regulate the production of proinflammatory cytokines in peripheral tissue inflammatory diseases, such as rheumatoid arthritis, is the p38 mitogen activated protein kinase (MAPK) family [11,12]. The p38 MAPK family consists of at least four isoforms (p38α, β, δ, γ), which are encoded by separate genes, expressed in different tissues and have distinct functions [13]. Activation of p38 MAPK signaling has been shown to regulate gene expression and lead to increased production of proinflammatory cytokines by a number of different mechanisms [for review, see: [14]]. The p38 MAPK pathway has been suggested to play a central role in various pathological CNS conditions including cerebral ischemia [15,16] and Parkinson's disease [17-19], as well as in AD [20,21], where postmortem studies find p38 MAPK activation occurs at the very early stage of the disease [20,22].

Previously we have shown using both a pharmacological approach with a selective small molecule p38α MAPK inhibitor and a genetic approach with primary microglia that are deficient in p38α that the α isoform of p38 MAPK is critical for the production of IL-1β and TNFα from activated microglia [23]. Moreover, suppression of p38α MAPK with the small molecule inhibitor in an AD-relevant mouse model was also found to decrease brain proinflammatory cytokine production, and attenuate synaptic protein loss [24]. These data suggested that microglia p38α MAPK is critical to inflammation-induced neurotoxicity. In the current study, we explored whether there is a causative link between microglia p38α MAPK signaling and neuronal damage, as well as a potential mechanism for microglia-dependent neurotoxicity. We used primary microglia from either wild-type (WT) mice or from p38α MAPK conditional knockout (KO) mice in co-culture with cortical neurons from WT mice. In WT microglia/neuron co-cultures, LPS treatment led to a significant increase in TNFα production, loss of synaptic proteins, and neuronal death. Neurons in co-culture with p38α-deficient microglia showed reduced LPS-induced TNFα production and were protected against synaptic loss and neuronal death. The mechanism of neurotoxicity was explored by showing that addition of a neutralizing TNFα antibody prevented neuronal degeneration in WT microglia-neuron co-cultures, and addition of recombinant TNFα to KO microglia-neuron co-cultures led to enhanced neuronal degeneration. Our data support the conclusion that activation of p38α MAPK and the downstream overproduction of the proinflammatory cytokine TNFα play a major role in the dysregulated microglial response to LPS that leads to neuron degeneration.

## Results

### Validation of microglia p38α MAPK deletion in conditional KO mice

**Figure 1:**
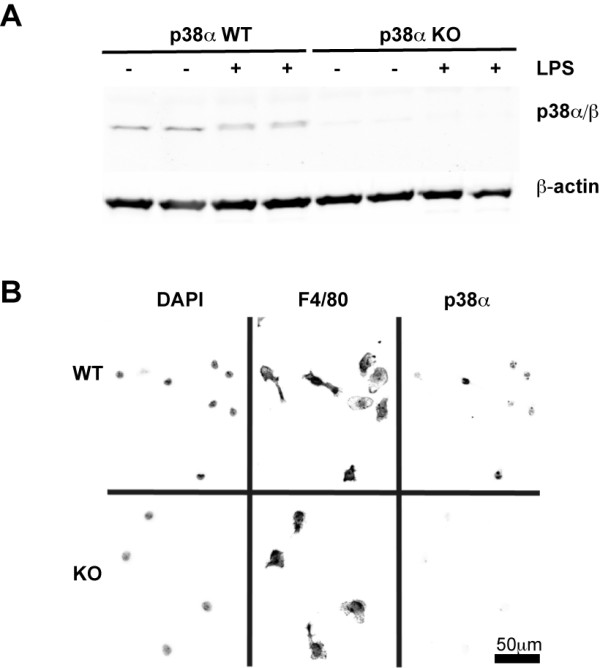
**Validation of microglia p38α MAPK deletion in conditional KO mice**. Microglia were plated at a density of 1 × 10^5 ^cells in 24-well plates for immunoblotting (A) and 5 × 10^3 ^cells onto 12-mm glass coverslips for immunocytochemistry (B). Microglia from p38α wild-type (WT) mice show clear bands for p38α/β MAPK, but essentially no p38α/β was seen in microglia from p38α knockout (KO) mice by either methodology, confirming the deficiency of p38α in the KO microglia. No obvious difference in the morphology of microglia was observed between the p38α WT and p38α KO.

In the CNS, p38α MAPK is not restricted to microglia; therefore, to determine the importance of p38α MAPK specifically in microglia, we used primary microglia isolated from p38α conditional KO mice, where p38α is genetically deficient in microglia [25]. Microglia isolated from mice with the loxP-flanked p38α allele but not carrying the Cre allele (p38α WT) were found to have levels of p38α MAPK similar to microglia from C57BL/6 mice (data not shown). We confirmed that this conditional gene deletion approach was highly efficient at eliminating the levels of p38α MAPK from microglia as determined by immunoblotting. Specifically, microglia isolated from p38α KO mice showed essentially no p38α compared to the p38α WT microglia cells, either under control conditions or after treatment with LPS (Figure 1A). In addition, we confirmed the absence of p38α in the microglia cultures from KO mice by immunocytochemistry (Figure 1B).

The absence of p38α MAPK did not affect the number of microglia that were isolated from the p38α KO mice. It also did not affect the overall morphological appearance of the microglia in culture. For example, there was no obvious difference in the morphology of microglia in the p38α KO group compared to the p38α WT group, as demonstrated by the microglia marker F4/80 (Figure 1B). These results were confirmed with two additional microglia-specific markers IBA1 and CD11b (data not shown). These data also documented that the microglia isolation method used resulted in a highly enriched population of microglia, with essentially > 99% purity for both the p38α WT and KO microglia cells.

### Microglial p38α MAPK deficiency prevents LPS-induced neurotoxicity in microglia/neuron co-cultures

**Figure 2:**
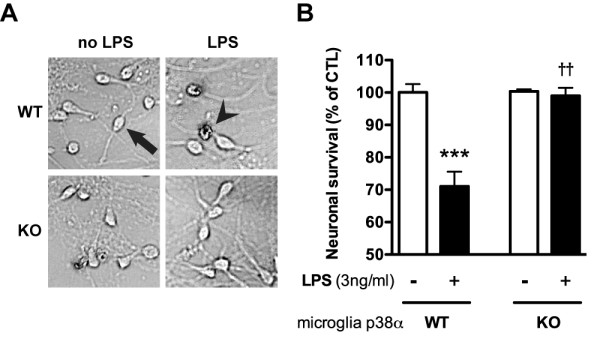
**Microglial p38α MAPK deficiency prevents LPS-induced neurotoxicity in microglia/neuron co-cultures**. Primary cortical neurons were co-cultured with primary microglia from p38α WT or p38α KO mice. The microglia/neuron co-cultures were stimulated with 3 ng/ml LPS for 72 h, then neurons analyzed by trypan blue exclusion assay. Representative photomicrographs of the neuron cultures are shown in (A). The arrow indicates a live neuron typical of the non-LPS stimulated co-culture. The arrowhead shows a dead neuron that is positive for trypan blue. In microglia from p38α WT mice, LPS produced a significant decrease in neuronal survival (B; ***p < 0.001). No significant neuron death was seen in microglia from p38α KO mice stimulated with LPS. Data represents 3 independent experiments.

Activated microglia are capable of secreting bioactive molecules, such as reactive oxygen and reactive nitrogen species, as well as proinflammatory cytokines, all of which have the potential to be neurotoxic [7]. We have previously implicated p38α MAPK signaling as important for glia-induced neuronal death in a mixed glia/neuron co-culture system [26]. However, p38α MAPK is present in multiple CNS cell types, including microglia, astrocytes, and neurons. Therefore, in this study, we took a different approach to determine the specific contribution of microglia p38α MAPK to glia-induced neuronal death. Specifically, we isolated microglia from either p38α KO or p38α WT mice, placed them in co-culture with WT primary cortical neurons, and tested whether the absence or presence of microglia p38α would affect LPS-induced neurotoxicity. Consistent with what we previously reported [26], LPS had no effect on neuronal viability in the absence of microglia (100 ± 1.3; 93.4 ± 7.7; % survival without and with LPS, respectively). Also as expected, treatment of WT microglia/neuron co-cultures with LPS for 72 h led to significant neuronal death, as determined by trypan blue exclusion assay (Figure 2). In contrast, WT neurons co-cultured with p38α KO microglia were resistant to LPS-induced neurotoxicity, showing essentially 100% survival (Figure 2).

### Microglial p38α MAPK deficiency attenuates LPS-induced synaptic protein loss in microglia/neuron co-cultures

**Figure 3:**
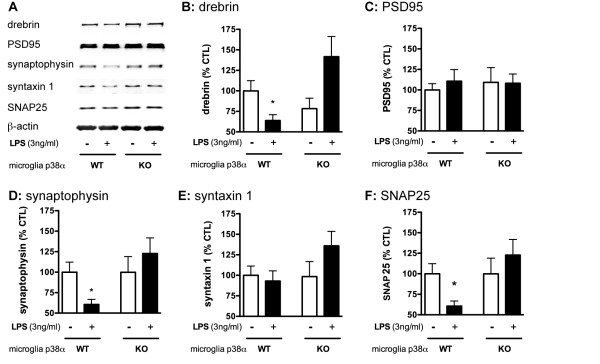
**Microglial p38α MAPK deficiency attenuates LPS-induced synaptic protein loss in microglia/neuron co-cultures**. Mouse primary neurons (5 × 10^4^) were co-cultured with either p38α WT microglia or p38α KO microglia (2 × 10^4^) in the absence or presence of LPS (3 ng/ml) for 72 h. Neuronal lysates were analyzed by immunoblotting; a representative blot is shown in (A). The levels of the post-synaptic protein drebrin (B) but not PSD95 (C) were significantly decreased by LPS exposure in p38α WT microglia. However, LPS treatment of the p38α KO microglia/neuron co-cultures did not produce a significant decrease in drebrin or PSD95. (D) LPS treatment of p38α WT microglia/neuron co-cultures produced a significant decrease in synaptophysin levels, which was not seen in LPS stimulated co-cultures of p38α KO microglia/neurons. (E) Levels of syntaxin 1 were unaffected by LPS in either WT or KO microglia. (F) Levels of SNAP25 were significantly decreased in the LPS-stimulated p38α WT microglia co-cultures, but not in microglia co-cultures from p38α KO mice. (*p < 0.05; p38α WT vs. p38α WT+LPS). Data represents 2-3 independent experiments.

We used the microglia/neuron co-culture system to address whether secreted factors from activated microglia can produce synaptic changes in the neurons and whether microglia p38α plays a role in these responses. We measured protein levels by immunoblotting (Figure 3A) for a panel of five synaptic proteins: two postsynaptic proteins, drebrin (Figure 3B), and PSD95 (Figure 3C); and three presynaptic proteins, synaptophysin (Figure 3D), syntaxin 1 (Figure 3E), and SNAP25 (Figure 3F). As an initial control, we measured PSD95 and synaptophysin levels in neuronal cultures treated with LPS for 72 h in the absence of microglia, and confirmed no effect on these synaptic proteins (% PSD95 levels: 100 ± 9.2 and 94.5 ± 8.6, without and with LPS respectively; % synaptophysin levels: 100 ± 10.6 and 95.9 ± 1.6, without and with LPS respectively). However, when LPS was added to the WT microglia/neuron co-culture (Figure 3), there was a significant decrease in three of the five synaptic proteins measured: namely, drebrin, synaptophysin, and SNAP25. Microglia p38α is involved in these responses, as demonstrated by the observation that the absence of microglia p38α protected against the LPS-induced decrease in drebrin, synaptophysin and SNAP25. There were no significant LPS-induced changes in levels of PSD95 or syntaxin 1 in co-cultures with either WT microglia or p38α KO microglia.

### Microglial p38α MAPK-dependent TNFα is involved in LPS-induced neuronal death

**Figure 4:**
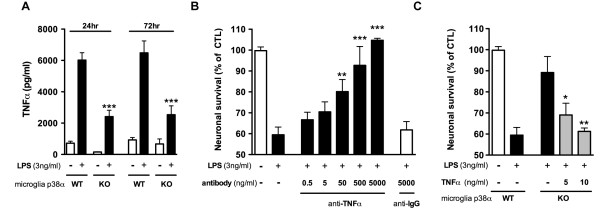
**Microglial p38α MAPK-dependent TNFα is involved in LPS-induced neuronal death**. (A) TNFα levels in the conditioned media were measured at 24 h and 72 h after LPS addition. The TNFα response to LPS was significantly reduced in the p38α KO microglia/neuron co-cultures (***p < 0.0005; p38α WT+LPS vs. p38α KO+LPS). (B) Addition of a neutralizing antibody to TNFα in p38α WT microglia/neuron co-culture abolished LPS-induced neurotoxicity in a concentration-dependent manner, with significant protective effects at concentrations of 50 ng/ml and higher. The non-immune rabbit IgG control antibody at 5000 ng/ml had no protective effect (**p < 0.005 or ***p < 0.0005; compared to p38α WT+LPS; Bonferroni's multiple comparison test). (C) Addition of exogenous TNFα (5 ng/ml or 10 ng/ml) to the LPS-stimulated p38α KO microglia reduced neuronal survival. Compared to the p38α KO microglia stimulated with LPS alone, 5 ng/ml (*p < 0.05) and 10 ng/ml (**p < 0.005) TNFα significantly decreased neuronal survival (Bonferroni's multiple comparison test). Data represents 3-6 independent experiments.

Activated microglia can produce a variety of secreted molecules that have the potential to be neurotoxic, including proinflammatory cytokines such as TNFα. We have previously reported [23] that the production of TNFα from activated microglia is dependent on the p38α MAPK pathway. Therefore, this cytokine was a logical candidate to test for involvement in the microglia-induced neurotoxicity seen in our co-cultures. We first determined if LPS-treated microglia/neuron co-cultures are associated with elevated TNFα level. In neurons cultured alone, with or without LPS, TNFα was below the limit of detection (< 3.4 pg/ml). In WT microglia/neuron co-cultures, LPS stimulated a ~6-fold increase in TNFα levels (Figure 4A). The levels of TNFα reached maximum after 24 h of LPS treatment and remained high until the 72 h time-point (data for 48 h not shown). In co-cultures with p38α KO microglia stimulated with LPS, the levels of TNFα were significantly (p < 0.0005) less than in co-cultures with WT microglia at all three time-points.

We next addressed the question of whether TNFα overproduction is essential for the neurotoxicity observed in the microglia/neuron co-cultures. To test this hypothesis, we used a TNFα neutralizing antibody to decrease the TNFα levels in WT microglia/neuron co-culture. As shown in Figure 4B, LPS caused ~40% neuronal death. When a TNFα neutralizing antibody was added to the culture, we found a concentration-dependent neuroprotection. At a concentration of 50 ng/ml or higher of the neutralizing antibody there was a significant reduction in LPS-induced neuronal death, reaching 100% neuronal survival at 5 μg/ml anti-TNFα antibody. In contrast, the administration of non-immune isotype control antibody (5 μg/ml) failed to protect neurons from the LPS-induced neuronal death (Figure 4B). These results suggest that blocking TNFα in WT microglia/neuron co-cultures is sufficient to prevent LPS-induced neuronal death.

As a complementary approach to determining the involvement of TNFα in the LPS-induced neurotoxicity, we tested whether the enhanced neuronal survival seen in p38α KO microglia/neuron co-cultures could be influenced by adding back TNFα to levels seen in WT microglia/neuron co-cultures. As shown in Figure 4A, in the p38α KO microglia/neuron co-cultures treated with LPS, TNFα is decreased on average ~5 ng/ml compared to WT. Therefore, two concentrations of TNFα (5 and 10 ng/ml) were administered along with LPS to the p38α KO microglia/neuron co-cultures, and neuronal survival was measured after treatment for 72 h. At a concentration of 5 ng/ml or 10 ng/ml TNFα, we found a significant concentration-dependent increase in neuronal death compared to the p38α KO microglia/neuron co-cultures that were stimulated with LPS alone (Figure 4C). These results demonstrate that addition of TNFα to p38α KO microglia/neuron co-cultures increases LPS-induced neurotoxicity to levels comparable to that seen in WT microglia/neuron co-cultures.

### Microglial p38α MAPK-dependent TNFα is involved in LPS-induced neurite degeneration

**Figure 5:**
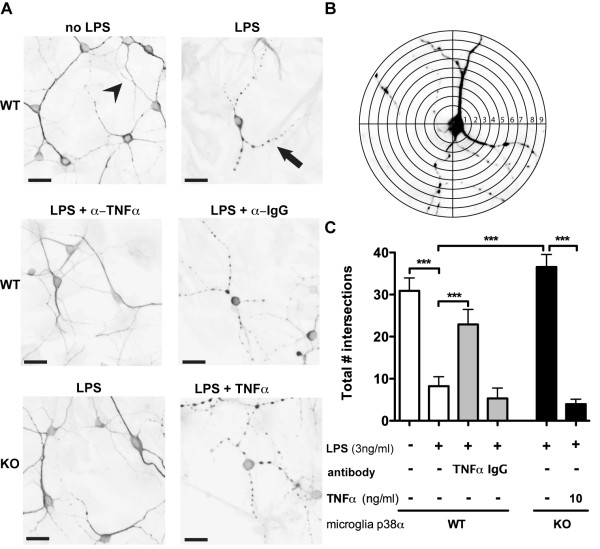
**Microglial p38α MAPK-dependent TNFα is involved in LPS-induced neurite degeneration**. (A) Photomicrographs of MAP-2 immunocytochemistry show the morphology of neurons after 72 h of co-culture with microglia. The arrow points to the appearance of neurites that have been damaged by LPS-activated WT microglia. In contrast, the arrowhead points to the morphological appearance of healthy, undamaged neurites. (B) Diagram of the Sholl method for quantifying the total number of healthy neurites that intersect the concentric circles. (C) Quantification of healthy neurites by the Sholl analysis demonstrates that LPS stimulation of p38α WT microglia in co-culture causes neurite degeneration as seen by a significant reduction in the number of intersections by healthy neurites in the LPS-stimulated group compared to the unstimulated group (white bars). This degeneration can be attenuated by the addition of a blocking antibody to TNFα (5 μg/ml), while the non-immune IgG control was not protective (gray bars). Microglia from p38α KO mice stimulated with LPS (black bar) also have significantly less neurite degeneration than the LPS-stimulated p38α WT microglia (white bar). However, by adding TNFα back to the p38α KO microglia co-culture, there is a significant decrease in the healthy neurite arborization compared to the p38α KO microglia stimulated with LPS alone (black bars). (***p < 0.005; Bonferroni's multiple comparison test). Data represents 2 independent experiments. Scale bar equals 25 μm.

Following LPS stimulation of microglia/neuron co-cultures we found, by immunocytochemistry for MAP-2, that neurites of surviving neurons had marked swellings, with an appearance of beads on a string (see arrow, Figure 5A). These swellings, or blebs, were not seen in co-cultures without LPS stimulation (see arrowhead, Figure 5A). In order to quantify these observations, we used Sholl analysis [27] to quantify the total number of intersections that neurites made with the concentric circles (Figure 5B). We found no significant difference between the groups in terms of the total number of neurite intersections, irrespective of whether the neurite was smooth or had blebs (28.9 ± 2.74 average across groups). However, as shown in Figure 5C, when we quantified only the neurites that are smooth, indicative of a 'healthy' neurite, we found that LPS-stimulated WT microglia/neuron co-cultures showed a highly significant decrease in the healthy neurite arborization compared to the non-LPS-stimulated co-culture. Moreover, the degeneration of the neurites was dependent on p38α MAPK produced TNFα. This was demonstrated by a significant recovery in the numbers of healthy neurites either by addition of a TNFα blocking antibody to WT microglia or by p38α MAPK deficiency in microglia. We further found that adding back TNFα to the p38α KO microglia/neuron co-cultures recapitulated the neurodegenerative phenotype seen with the LPS-stimulated WT microglia.

## Discussion

In the current study, we used microglia/neuron co-cultures to document several important findings about the mechanisms by which activated microglia can produce neurodegenerative responses. First, the importance of microglia p38α MAPK signaling was demonstrated by the observations that neurons in co-culture with p38α-deficient microglia were protected against LPS-induced neurotoxicity, synaptic protein loss, and neurite degeneration. Second, p38α-dependent microglia TNFα production was shown to be involved in the mechanism of the LPS-induced neuron damage by the findings that p38α KO microglia produce much less TNFα in response to LPS compared to WT microglia, that adding back TNFα to p38α KO microglia increases the LPS-induced neurotoxicity, and that neutralization of TNFα in WT microglia decreases the LPS-induced neuron damage. Altogether, our results demonstrate the critical importance of the p38α MAPK signaling pathway and overproduction of the proinflammatory cytokine TNFα in the dysregulated microglia inflammatory responses to an LPS stressor, leading to microglia-induced neuronal dysfunction.

Our demonstration that microglia p38α MAPK signaling is important in the mechanism of LPS-induced neuron damage is consistent with numerous findings that have implicated p38 MAPK activation in the process of neuronal death in a variety of neurodegenerative disorders. In addition, our studies here using cell-selective, isoform-specific KO mice extend previous findings by showing that the p38α MAPK isoform in microglia is a key mediator of LPS-induced neuronal and synaptic dysfunction. We also provide evidence that one mechanism by which LPS activation of microglia p38α MAPK signaling leads to neuron death is through up-regulation of the proinflammatory cytokine TNFα.

The p38 MAPK family consists of four major isoforms (p38α, β, δ, γ) that have different cell and tissue expression patterns, substrate specificities, and functions [for reviews, see: [14,28]]. The patterns of expression and activation of the p38α isoform in peripheral immune cells [29,30] suggested that this isoform might play a major role in the inflammatory response. Early attempts using genetic KO approaches to explore the role of p38α in inflammatory responses were hampered because of embryonic lethality seen with global KO of p38α. However, a number of more recent studies have used conditional ablation of p38α in specific cell types to provide direct evidence that the p38α isoform is of central importance for many peripheral inflammatory responses, such as inflammation-induced arthritic bone loss [31], inflammatory skin injuries [13], inflammatory responses of myeloid cells in an experimental colitis model [32], immune cell recruitment and pathogen clearance in intestinal epithelial cells [33], and LPS-induced cytokine production in macrophages [25]. These and other studies using selective p38α inhibitors and drug-resistant forms of the kinase have demonstrated the importance of p38α signaling in mediating peripheral inflammatory responses [34-37].

Although there is broad agreement that p38α plays a key role in cytokine production and other inflammatory responses in peripheral immune cells, the contribution of p38α to pathological microglial activation and detrimental inflammation in CNS disorders is less well understood. Increasing evidence suggests that p38 signaling cascades contribute to CNS cytokine overproduction and neurodegenerative sequelae [for reviews, see: [14,38,39]], but few studies have tested the specific role of microglia p38α. Expression of the p38α isoform in microglia was reported to increase early after transient global ischemia [40], and administration of p38 inhibitors reduced infarct volume [15,41] and suppressed proinflammatory cytokine production [41]. We recently demonstrated [23] a direct linkage between microglia p38α and proinflammatory cytokine production in response to different stressors by showing that inhibition of p38α in microglia with either a pharmacologic or genetic approach suppresses proinflammatory cytokine up-regulation induced by toll-like receptor ligands or beta-amyloid.

In the present study, we explored the consequences of the microglial p38α-dependent proinflammatory cytokine response on neuronal endpoints. By using microglia deficient in p38α, we showed definitively that microglial p38α is critical for LPS-induced neuron dysfunction and we implicated p38α-dependent production of the proinflammatory cytokine TNFα in the mechanism of neuron damage. The potential involvement of TNFα was not unexpected, as this proinflammatory cytokine has been shown to induce neurotoxicity in models of CNS neurodegenerative disorders [42-44], and blocking TNFα signaling can be neuroprotective [45,46]. However, TNFα is pleiotropic and can also have neuroprotective functions [for review, see: [47]]. Multiple factors influence whether TNFα will exert neurotoxic or neuroprotective actions, including the level and duration of expression in a particular cell type or brain region, the microglia activation state, the particular disease or disease stage, the levels of different TNF receptors and adapter proteins, and the upstream activators and downstream effectors in the signaling pathways. Thus, it was somewhat surprising that microglia p38α-dependent production of TNFα in response to an LPS insult appeared to be sufficient to induce neuron death, as evidenced by the observations that anti-TNFα antibody treatment resulted in increased neuronal survival back to control values, and addition of TNFα to KO microglia reduced neuronal survival to the same levels as WT. Altogether, our data demonstrate that microglia p38α activation in response to an LPS stressor stimulus and the consequent dysregulated TNFα signaling can lead to neuron damage.

Of note is our finding that p38α MAPK deficiency in microglia attenuates LPS-induced loss of specific synaptic proteins in the co-cultures. Previous studies have shown a correlation between p38 MAPK activation and a decline in synaptophysin levels in AD transgenic mouse models and in primary microglia and cortical neuron co-cultures stimulated with LPS [48,49], and pharmacological inhibition of p38α MAPK significantly reduced TNFα and IL-1β production and prevented synaptophysin loss in an AD mouse model [24]. Our results here demonstrate for the first time a linkage of p38α MAPK and TNFα to LPS-induced decreases in SNAP25 and drebrin. Because drebrin, a postsynaptic protein found within dendritic spines, is important for spine morphogenesis and maintenance [50,51], future studies should examine in more detail the mechanisms by which p38α MAPK influences dendritic pathology and synaptic deterioration such as seen in many neurodegenerative disorders. Future studies should also explore whether microglia p38α MAPK is involved in beneficial responses of activated microglia, as the current study focused only on detrimental consequences of microglia p38α activation.

## Conclusions

We report that p38α MAPK in microglia plays a critical role in activated microglia-mediated neurotoxicity, loss of synaptic proteins, and neurite degeneration via a mechanism involving TNFα signaling. These results suggest that selective targeting of the p38α MAPK signaling pathway should be explored as a potential therapeutic strategy for the treatment of CNS disorders where overproduction of proinflammatory cytokines is implicated in disease progression.

## Methods

### Animals

All experiments were conducted in accordance with the principles of animal care and experimentation in the Guide For the Care and Use of Laboratory Animals. The Institutional Animal Care and Use Committee of the University of Kentucky approved the use of animals in this study. C57BL/6 mice were obtained from Harlan Laboratories. The p38α MAPK conditional knockout mice were generated as previously described [23,25], following a standard breeding scheme for conditional gene inactivation [52]. The first exon of the p38α gene (MAPK14) was flanked by two loxP sites. The mice were backcrossed to homozygosity so that both alleles of the p38α gene contained loxP sites (p38α^fl/fl^) and maintained on a C57BL/6 background. LysM-Cre mice expressing the Cre recombinase transgene under control of the lysozyme M promoter (B6.129-Lyzs^tm1(cre)Ifo/^J) were then crossed with the p38α^fl/fl ^mice. The LysMCre^+ ^p38α^fl/fl ^offspring were then crossed with the p38α^fl/fl ^mice to generate experimental and control animals. This generates litters where ~50% mice are p38α^fl/fl(+cre) ^(KO) and ~50% are p38α^fl/fl(-cre) ^(used as WT controls). The restricted cell-type expression of the lysozyme promoter [53,54] results in cell-specific deletion of p38α MAPK in myeloid cells including microglia. Genotyping was performed by Transnetyx, Inc (Cordova, TN).

### Primary neuronal culture

Primary neuronal cultures were derived from embryonic day 18, C57BL/6 mice, as previously described [26]. Briefly, cerebral cortices were dissected and the meninges were removed. Cells were dissociated by trypsinization (0.25% trypsin, 2.21 mM EDTA) for 15 min at 37°C and triturated, followed by passing through a 70 μm nylon mesh cell strainer. The neurons were plated onto poly-D-lysine-coated 12-mm glass coverslips at a density of 5 × 10^4^/well in 24 well plates. Neurons were grown in neurobasal medium (Invitrogen) containing 2% B27 supplement (Invitrogen), 0.5 mM L-glutamine, (Mediatech), and 100 IU/ml penicillin, 100 μg/ml streptomycin (Mediatech); no serum or mitosis inhibitors were used. Every 3 days, 50% of the media was replenished with fresh medium. The purity of the primary neuronal cultures was verified as 93% by immunocytochemistry for the neuronal marker NeuN, astrocyte marker GFAP, and microglia marker Iba-1 (data not shown).

### Microglia culture

Microglia cultures were prepared as previously described [23]. Briefly, mixed glial cultures (~95% astrocytes, ~5% microglia) were prepared from the cerebral cortices of 1-3 day old mice. The tissue was trypsinized as above, and the cells were resuspended in glia complete medium [α-minimum essential medium (α-MEM; Mediatech) supplemented with 10% fetal bovine serum (FBS) (US Characterized FBS; Hyclone; Cat no. SH30071.03), 100 IU/ml penicillin, 100 μg/ml streptomycin (Mediatech) and 2 mM L-Glutamine (Mediatech)]. After 10-14 days in culture, microglia were isolated from the mixed glial cultures by the shake-off procedure [55]. Specifically, loosely attached microglia were shaken off in an incubator shaker at 250 rpm for 2 h at 37°C, the cell-containing medium was centrifuged at 1100 rpm for 3 min, and the cells were seeded onto 12-mm glass coverslip at the density of 2 × 10^4 ^in 24 well plates, unless otherwise specified. Prior to plating the microglia on the coverslip, three equally spaced 1 mm glass beads (Borosilicate; Sigma) were attached to the coverslip with paraffin wax. The microglia cultures were verified to be > 99% microglia by immunocytochemistry. Microglia were incubated for one day before placing into co-culture with neurons.

### Primary neuron/microglia co-culture and cell treatments

Following previously described methods [26], after 7-9 days in culture, neurons on coverslips were co-cultured with mouse microglia by placing the microglia-containing coverslips cell side down into the neuron-containing wells. In this co-culture system, the microglia and neurons are in close apposition and share the same neurobasal/B27 culture media, but are separated by the 1 mm glass beads and do not have direct cell-cell contact. Lipopolysaccharide (LPS) from *Salmonella typhimurium *(Sigma) was resuspended in sterile saline at 100 mg/ml, and was used at a final concentration of 3 ng/ml for all experiments. A rat monoclonal IgG_1_, anti-mouse TNFα neutralization antibody (clone # MP6-XT22) with a reported 50% neutralization dose in the range of 0.15-0.75 μg/ml, was reconstituted in sterile PBS according to manufacturer specifications (R&D Systems). A rat IgG_1 _monoclonal antibody (clone # 43414) was used as a non-immune isotype control antibody (R&D Systems). Treatment with either antibody occurred 1 h prior to LPS treatment. Recombinant mouse TNFα (aa 80-235; R&D systems) was added at the same time as the LPS treatment.

### Neuronal Viability Assay

Neuron viability was assayed by trypan blue exclusion [26]. Neuron-containing coverslips were incubated with 0.2% trypan blue in Hanks' Balanced Salt Solution (HBSS) for 2 min in 37°C incubator and then rinsed 3 times with HBSS. Neurons were viewed under brightfield microscopy at 200× final magnification. Three to five fields were chosen per coverslip, and a total of 150 to 560 cells were counted per coverslip. Trypan blue-positive and negative neurons were counted per field and the ratio of negative cells to the total cells was taken as the index of neuronal survival rate.

### Immunocytochemistry

Cells were fixed with 3.7% formalin containing 0.1% Triton X-100 in PBS for 10 min at room temperature. After washing three times with PBS, the coverslips were incubated with blocking buffer (PBS containing 5% goat serum, 3% bovine serum albumin (BSA; Fisher Scientific), 0.1% Triton X-100) for 30 min at room temperature. Primary antibodies were diluted in blocking buffer and incubated with the cells at room temperature for 2 h. Primary antibodies used in this study were: chicken anti-MAP-2 antibody (1:100, Neuromics); mouse anti-NeuN (1:100, Millipore); rat anti-GFAP (1:1000, Invitrogen); rabbit anti-IBA1 (1:1000, Wako); rat anti-CD11b (1:100, Serotec); rat anti-F4/80 (1:100, Serotec); and p38α (1:100, R&D Systems). For detection of primary antibodies, species-appropriate Alexa Fluor^® ^fluorescent conjugated secondary antibodies (1:1000, Invitrogen) were incubated in blocking buffer at room temperature for 2 h. Wide field fluorescent photomicrographs were obtained using a Zeiss Axioplan 2 microscope with an Axiocam MRc5 digital camera (Carl Zeiss).

### Western blotting and ELISA assays

Western blotting was performed as previously described [55]. Briefly, whole cell lysates were prepared in sodium dodecyl sulfate (SDS)- containing sample buffer, and equal volumes of lysates were separated by 10.5-14% SDS-PAGE Criterion precast gel (Bio-Rad Laboratories). Proteins were transferred to nitrocellulose membrane using a dry blotting system (iBlot^® ^Invitrogen). Blots were probed using reagents and manufacturer recommendations for Odyssey Infrared Imaging system (LI-COR Biosciences), with the following primary antibodies: mouse anti-drebrin (1:5000, Abcam); rabbit anti-PSD95 (1:2000, Cell Signaling); mouse anti-synaptophysin (1:1000, Millipore); rabbit anti-syntaxin 1 (1:10, 000, Millipore), mouse anti-SNAP 25 (1:4000, BD Biosciences); rabbit anti-p38α/β (1:1000, Cell Signaling), and mouse anti-β-Actin (1:10, 000, Cell Signaling). Blots were visualized and analyzed on the Odyssey Infrared imaging system (LI-COR Biosciences), and integrated intensity values were used in statistics.

After 24 h, 48 h, and 72 h in the co-cultures, 20 μl conditioned medium was harvested for TNFα ELISA assay using kits from Meso Scale Discovery (MSD) according to the manufacturer's instructions.

### Sholl analysis

The Sholl method [27] was used in the quantification of MAP-2 labeled neurites. A series of concentric circles were drawn at 10 μm intervals starting with a diameter of 20 μm to a final diameter of 200 μm. Intersections of smooth or blebbed neurites with the concentric circles were counted. The total number of intersections for each neuron was plotted as a measure of neurite arborization. Per experimental condition, 20-30 neurons were analyzed from two independent experiments by an observer blinded to treatment conditions.

### Statistics

Statistical analysis was conducted using GraphPad prism software V.5 (GraphPad Software, La Jolla, CA). Unless otherwise indicated, values are expressed as mean ± SEM. Groups of two were compared by unpaired *t*-Test. One-way ANOVA followed by Bonferroni's multiple comparison test was used for comparisons among three or more groups. Statistical significance was defined as p < 0.05.

## List of abbreviations

(AD): Alzheimer's disease; (KO): knockout; (WT): wild-type; (LPS): lipopolysaccharide; (MAPK): mitogen-activated protein kinase; (TNF): tumor necrosis factor

## Competing interests

The authors declare that they have no competing interests.

## Authors' contributions

BX, ADB and LVE designed the studies. BX performed the experiments in cell culture. BX and ADB performed the data analysis. BX and ADB jointly drafted the manuscript together with LVE. All authors read and approved the final version. BX and ADB contributed equally to this study.
